# Three-Dimensional Disassemblable Scaffolds for Breast Reconstruction

**DOI:** 10.3390/polym17152036

**Published:** 2025-07-25

**Authors:** Viktoriia Kiseleva, Aida Bagdasarian, Polina Vishnyakova, Andrey Elchaninov, Victoria Karyagina, Valeriy Rodionov, Timur Fatkhudinov, Gennady Sukhikh

**Affiliations:** 1 National Medical Research Center for Obstetrics, Gynecology and Perinatology Named after Academician V.I. Kulakov of Ministry of Healthcare of Russian Federation, Moscow 117997, Russia; 2 Research Institute of Molecular and Cellular Medicine, Peoples’ Friendship University of Russia (RUDN University), Moscow 117198, Russia; 3 Avtsyn Research Institute of Human Morphology of Federal State Budgetary Scientific Institution “Petrosky National Research Centre of Surgery”, Moscow 117418, Russia

**Keywords:** scaffolds, reconstruction, breast cancer

## Abstract

In recent years, significant progress has been made in breast reconstructive surgery, particularly with the use of three-dimensional (3D) disassemblable scaffolds. Reconstructive plastic surgery aimed at restoring the shape and size of the mammary gland offers medical, psychological, and social benefits. Using autologous tissues allows surgeons to recreate the appearance of the mammary gland and achieve tactile sensations similar to those of a healthy organ while minimizing the risks associated with implants; 3D disassemblable scaffolds are a promising solution that overcomes the limitations of traditional methods. These constructs offer the potential for patient-specific anatomical adaptation and can provide both temporary and long-term structural support for regenerating tissues. One of the most promising approaches in post-mastectomy breast reconstruction involves the use of autologous cellular and tissue components integrated into either synthetic scaffolds—such as polylactic acid (PLA), polyglycolic acid (PGA), poly(lactic-co-glycolic acid) (PLGA), and polycaprolactone (PCL)—or naturally derived biopolymer-based matrices, including alginate, chitosan, hyaluronic acid derivatives, collagen, fibrin, gelatin, and silk fibroin. In this context, two complementary research directions are gaining increasing significance: (1) the development of novel hybrid biomaterials that combine the favorable characteristics of both synthetic and natural polymers while maintaining biocompatibility and biodegradability; and (2) the advancement of three-dimensional bioprinting technologies for the fabrication of patient-specific scaffolds capable of incorporating cellular therapies. Such therapies typically involve mesenchymal stromal cells (MSCs) and bioactive signaling molecules, such as growth factors, aimed at promoting angiogenesis, cellular proliferation, and lineage-specific differentiation. In our review, we analyze existing developments in this area and discuss the advantages and disadvantages of 3D disassemblable scaffolds for mammary gland reconstruction, as well as prospects for their further research and clinical use.

## 1. Introduction

Breast cancer (BC) is one of the most pressing issues in modern oncology, primarily due to its high morbidity rate. According to The Global Cancer Observatory: CANCER TODAY (GLOBOCAN), a joint project of the World Health Organization (WHO) and the International Agency for Research on Cancer (IARC), more than 2.2 million new BC cases were reported worldwide in 2022 [1]. In the Russian Federation, 81,784 new cases of BC were identified in 2023 [2]. At the same time, BC ranks first among overall oncological morbidities in our country, accounting for 12.1%. For many years, this tumor has been the leading cause of female oncological morbidity, reaching 22.1%.

Surgery remains the main method of BC treatment [3]. The mammary gland is an aesthetically significant organ for women. At the same time, mastectomies account for up to 60% of surgical interventions for breast cancer. Complete removal of the mammary gland leads to physical trauma and deep emotional distress, causing deterioration of personal and family relationships. Reconstructive plastic surgeries aimed at restoring the shape and size of the mammary gland provide medical, psychological, and social rehabilitation, hereby improving their quality of life. Currently, breast reconstruction is performed using implants or autologous tissues. Reconstruction with allomaterials is the leading method of restoring the mammary gland after mastectomy.

Thus, according to the American Society of Plastic Surgeons, 65% of mammary gland reconstructions use implants [4]. In Europe, including Russia, various authors report that this percentage is as high as 80% [5].

The active use of silicone endoprostheses is facilitated by several advantages: a shorter operation time compared to methods using the patient’s own tissues, no need to collect donor tissue, a simple surgical technique, less trauma, reduced hospital stays, and faster postoperative patient rehabilitation. However, it should be noted that, within five years of reconstruction with alloplastic implants, 40% of patients face repeated operations due to early complications (infection, hematoma, seroma, asymmetry, or implant extrusion) or late complications (capsular contracture or implant rupture). Moreover, a drawback of implants is the potential for secondary tumors, such as anaplastic large cell lymphoma [6].

Using autologous tissues enables surgeons to recreate the appearance and tactile sensations of a healthy mammary gland while avoiding complications associated with foreign objects. However, this reconstruction method is more complex, labor-intensive, and expensive; it also damages the donor tissue area. Due to the challenges posed by implant-based and autologous tissue reconstruction, new approaches to breast reconstruction must be explored. This review critically examines current advancements in 3D disassemblable scaffold technologies for breast reconstruction, analyzing their design principles, clinical applications, and future potential. For a more in-depth analysis of the concepts and techniques of alloplastic (implant-based) breast reconstruction, we recommend reading the review by Frey and colleagues [7]. The principles of cell and tissue engineering can be applied to recreate major types of human body tissues: hard tissues (such as bone and cartilage), soft tissues (for example, skin), sensory tissues (nerve tissue), etc. Despite significant differences in the structure of tissues belonging to different categories, the principles of tissue engineering for each type have common elements. To regenerate any particular tissue, it requires tissue-forming cells, appropriate matrix, and growth factors ensuring proper functioning of the cells [8]. It is difficult to overestimate the role of selecting a suitable scaffold when creating various tissue-engineered constructs. Firstly, the scaffold determines the mechanical properties of the resulting construct. For instance, during stimulation of skin regeneration, it is specifically the matrix that ensures the layered structure characteristic of skin [9]. Hydroxyapatite and calcium phosphate-based scaffolds provide mechanical support and stimulate osteogenesis [10]. Materials intended for nerve regeneration should effectively promote directed axon growth. For this purpose, PLGA-based scaffolds combined with neurotrophic factors are commonly used [11]. For mammary gland tissue engineering, restoration of organ volume and its mechanical properties are of crucial importance [12].

## 2. Definition of Breast Reconstruction Methods

Before starting a discussion of manufacturing technologies, biological compatibility, preclinical and clinical applications of three-dimensional scaffolds for breast reconstruction, it is necessary to introduce definitions of what the authors understand/mean by the definitions of “traditional methods” and “3D disassemblable scaffolds”.

To date, traditional methods of breast reconstruction are well-established surgical techniques whose main purpose is to restore the shape and appearance of the breast after mastectomy. That can be divided into two main categories, including reconstruction using implants and transplantation of autologous tissues (autotransplantation) [13,14]. The use of synthetic implants consisting of silicone, acellular dermal matrix (ADM), etc., is the basis of the first group of breast reconstruction methods [15]. The second group of methods uses the body’s own tissues, such as fat, skin, and sometimes muscle, to restore breast shape. It is worth noting that in some cases in the literature, lipofilling is classified as a separate group of breast reconstruction methods. Table 1 presents the key points characterizing traditional breast reconstruction methods. The use of silicone implants dates back to the mid-20th century [14]. The safety of their use has been shown in several clinical studies (Table 1) [16,17,18,19]. The most common complications linked to breast reconstruction with silicone gel implants are capsular contracture, implant rupture, infection, seroma, and the need for reoperation [20]. In rare cases, the use of silicone implants has led to the development of breast implant-associated anaplastic large cell lymphoma (BIA-ALCL) [17]. In Russia, 70% of all breast reconstructive surgeries after mastectomy use the first group of methods [21]. At the same time, among the methods using autologous tissues, DIEP is a commonly used technique [22].

The use of autologous tissues for breast reconstruction is the technology of choice for patients with sufficiently developed volumes of muscles and fat on the abdomen, back, or hips [23]. However, the use of the body’s own tissues reduces the risk of complications associated with the introduction of artificial implants, but increases the incidence of necrosis of adipose tissue, the development of seromas, as well as aesthetic problems in the form of scars, tissue withdrawal, asymmetry of the hips, weakening of the back muscles, etc. Significant disadvantages of breast reconstruction methods include the high cost (ADMs add USD 2500–5000 per breast to procedure costs) and the lack of data on the long-term effects of use, which are also based on autologous tissues.

Seroma development is a common complication of breast reconstruction surgery, with reported rates ranging from 15% to 85% [24].

**Table 1:** Types and key features of traditional breast reconstruction techniques.

Method	Material	Principle of the Method	National Clinical Trial (NCT)	Advantages	Disadvantages
Implant-based reconstruction	Silicone Gel Implants	The implant is a silicone elastomer shell filled with a cohesive silicone gel that is FDA-approved for breast reconstruction, mimicking the natural feel of breast tissue. They are available in a variety of shapes (round or anatomical) and textures (smooth or textured). Example: Mentor MemoryGel (Mentor Worldwide LLC, Netherlands)	Mentor— NCT00812097 NCT00756652 NCT01009008 NCT02724371 Motiva [20]— NCT06274736 NCT05459064	Shorter surgery time compared to autologous tissue reconstruction. No donor-site morbidity (unlike flap-based reconstructions). Improved natural feel compared to saline implants (due to cohesive gel). Long-term FDA-approved safety data in multiple clinical studies.	Risk of capsular contracture (scar tissue hardening around the implant). Potential for implant rupture or leakage (though modern implants are more durable). Need for future revisions (implants are not lifetime devices; average lifespan ~10–20 years). Less natural movement compared to autologous tissue reconstruction. Possible association with BIA-ALCL (Breast Implant-Associated Anaplastic Large Cell Lymphoma)—rare but linked to textured implants.
	Saline Implants	Filled with sterile saline solution; less natural feel but adjustable in volume. Modern type is a structured saline implant, which is also filled with sterile salt water, but is made with an inner structure to help give the reconstructed breast a more natural look and feel.	Natrelle [25] NCT00691327 NCT00689871 NCT01870869 NCT01785069 NCT01853605 NCT00690339	Safety: If ruptured, saline is harmlessly absorbed by the body (unlike silicone gel leakage). Smaller incisions: Implants are inserted empty and then filled, requiring a smaller surgical opening. Adjustable volume: Surgeons can fine-tune size during surgery for better symmetry. Lower cost: Generally, less expensive than silicone gel implants. No association with BIA-ALCL (Breast Implant-Associated Anaplastic Large Cell Lymphoma).	Less natural feel: Firmer and more prone to rippling/wrinkling compared to silicone. Higher risk of deflation: Rupture leads to immediate volume loss (vs. silicone’s “silent rupture”). Lower long-term patient satisfaction: Often perceived as less natural-looking than silicone. More visible edges: Especially in thin patients with minimal soft-tissue coverage.
	Tissue expanders	Tissue expanders (TEs) are temporary, adjustable implants used in staged breast reconstruction to gradually stretch the skin and muscle to create a pocket for a permanent breast implant or autologous flap. They consist of a silicone shell with an integrated or remote fill port, allowing controlled saline injections over weeks to months.	NCT01222390 NCT01903174	Customizable expansion: Allows gradual stretching of skin/muscle, reducing tension and complications. Preserves options: Can be used before implant-based or autologous reconstruction. Lower initial morbidity: Less invasive than immediate flap reconstruction. Improved symmetry: Adjustable fill optimizes breast mound shape before permanent implant placement. Compatible with radiation therapy: Expanders can be left inflated during radiotherapy, delaying final reconstruction until tissue stabilizes.	Requires multiple procedures: Two-stage process (expansion → implant exchange). Discomfort during expansion: Temporary pain/pressure during saline fills. Risk of complications: Infection (5–15% risk, higher in irradiated patients). Capsular contracture (10–20% risk, increased with radiotherapy). Expander exposure/extrusion (rare but serious). Temporary asymmetry: During expansion phase.
	Acellular Dermal Matrix (ADM) [26]	Acellular Dermal Matrix (ADM) is a biologically derived scaffold (human, porcine, or bovine) processed to remove cellular components while preserving the extracellular matrix. Human-derived: AlloDerm^®^ (LifeCell), FlexHD^®^ (MTF). Porcine-derived: Strattice™ (AbbVie), XenMatrix™ (Bard), FORTIVA Porcine Dermis (USA). Bovine-derived: SurgiMend^®^ (TEI Biosciences).	NCT06456554 NCT04661501 NCT00872859 NCT06575192 NCT06555692 AlloDerm NCT01561287 NCT03064893 NCT04710537 NCT01781299 Strattice NCT00619762 NCT02521623 NCT02608593 SurgiMend NCT02521623 FORTIVA NCT03744013	Improved Aesthetic Outcomes: Creates a natural inframammary fold and lower pole projection. Reduces implant visibility/rippling (especially in thin patients). Facilitates Single-Stage Reconstruction: Enables direct-to-implant (DTI) reconstruction in select patients. Supports Prepectoral Placement: Allows muscle-sparing techniques, reducing animation deformity. Reduced Capsular Contracture Rates: Studies show lower rates vs. submuscular-only placement (e.g., 8% vs. 20%).	Higher Cost: ADM adds USD 2500–5000 per breast to procedure costs. Risk of Complications: Seroma (5–15%), infection (3–10%), delayed healing. Learning Curve: Requires precise handling (hydration, orientation) to avoid complications. Limited Long-Term Data: Durability beyond 10 years is not well-studied.
Breast shape restoration using autologous tissues	TRAM Flap Transverse Rectus Abdominus Myocutaneous	Used a portion of the lower abdominal skin, fat, and rectus abdominis muscle. There are two types based on blood supply: -Pedicle TRAM: the flap rests on the superior epigastric artery (the muscle remains attached) -Free TRAM: The flap is completely detached and reconnected using microsurgery to the thoracodorsal/internal thoracic vessels.	NCT00500565	Uses the patient’s own tissue, mimicking natural breast consistency. No risk of implant rupture or capsular contracture. Removes excess abdominal skin/fat (“tummy tuck” benefit). Avoids complications associated with implants (e.g., infection, rejection).	Donor-Site Morbidity: Abdominal Weakness/Hernia: Due to rectus muscle harvest (up to 5% risk). Bulging or Asymmetry: From muscle sacrifice. Longer Surgery/Recovery: Compared to implant-based reconstruction. Fat Necrosis: Partial flap loss due to inadequate blood supply (5–15% risk). Not Suitable for All Patients: Thin patients or those with prior abdominal surgeries may not qualify.
	DIEP Flap Deep Inferior Epigastric Perforator	Used skin and fat from the lower abdomen. Blood Supply: Deep inferior epigastric perforator vessels (microsurgically reconnected to chest vessels).	NCT00514748 NCT05363189 NCT00543764 NCT03481140 NCT05764577 NCT01398982 NCT00543907 NCT01469494	Muscle Preservation: No muscle sacrifice → lower risk of abdominal bulging/hernia vs. TRAM flap. Natural, Long-Lasting Results: Autologous tissue mimics natural breast aging. Reduced Donor-Site Morbidity: Faster recovery than TRAM, with less postoperative pain. No Implant Risks: Eliminates concerns about rupture, capsular contracture, or infection.	Technically Demanding: Requires microsurgical expertise (higher risk of flap failure if vessels are damaged). Longer Surgery Time: ~4–8 h vs. 2–3 h for implant-based reconstruction. Fat Necrosis Risk: 5–15% risk if blood supply is compromised. Not Suitable for Very Thin Patients: Insufficient abdominal tissue may necessitate alternative flaps (e.g., SGAP).
	Latissimus Dorsi Flap	Used a pedicled flap of skin, fat, and the latissimus dorsi muscle from the upper back. It is often combined with a breast implant to provide sufficient volume. Blood Supply: Thoracodorsal artery (remains attached as a pedicle, eliminating the need for microsurgery).	NCT03106233 NCT02442401 NCT06319157	Reliable Blood Supply: Pedicled flap reduces risk of total flap failure compared to free flaps. No Microsurgery Needed: Simpler than DIEP or free TRAM flaps. Useful for Radiation-Damaged Tissue: Provides well-vascularized coverage over implants in irradiated patients. Consistent Donor Site: Suitable for thin patients who lack abdominal tissue.	Donor-Site Morbidity: Back Scar: Horizontal or oblique scar on the back. Shoulder Weakness: Temporary reduced shoulder strength (improves with rehab). Often Requires an Implant: LD flap alone may lack sufficient volume (50–70% of patients need an implant). Seroma Risk: Up to 30% risk of seroma formation at the donor site.
	SGAP/IGAP Flap Superior/Inferior Gluteal Artery Perforator Flap	Used skin and fat from the buttocks while underlying the gluteal muscle. Blood Supply: Superior (SGAP) or inferior (IGAP) gluteal artery perforators.		No Abdominal Weakness: Preserves rectus and gluteal muscles (unlike TRAM flaps). Natural, Durable Results: Autologous fat mimics natural breast aging. Alternative for Thin Patients: Ideal when abdominal tissue is insufficient. Hidden Donor-Scar: Scar is concealed under clothing (bikini line for IGAP).	Technically Challenging: Short pedicle length and difficult dissection of perforators. Longer Surgery Time: ~6–8 h due to microsurgical complexity. Donor-Site Contour Irregularities: Risk of buttock asymmetry or depression. Higher Fat Necrosis Rates: Up to 15% due to variable perforator anatomy.
	TUG Flap Transverse Upper Gracilis (TUG) flap	Used a skin and fat paddle from the inner thigh, along with the gracilis muscle. The TUG flap is based on the medial circumflex femoral artery (MCFA), the dominant pedicle supplying the gracilis muscle and overlying subcutaneous tissue. The flap is harvested in a transverse orientation along the upper inner thigh, resulting in a well-concealed scar.		Alternative for patients with inadequate abdominal tissue (e.g., thin patients or those with prior abdominal surgeries). Favorable donor-site scar (hidden in the groin crease). Minimal functional morbidity (the gracilis muscle is a non-essential adductor). Suitable for bilateral breast reconstruction (both thighs can be used).	Limited volume (best for small to moderate-sized breast reconstructions). Donor-site complications (e.g., wound dehiscence, seroma, scar widening, or tightness in the thigh). Shorter pedicle length (~6–8 cm) compared to DIEP flaps, making microsurgical anastomosis more challenging. Potential for sensory changes in the inner thigh.
	Lipofilling, also called autologous fat grafting or fat transfer	Involves harvesting a patient’s own fat from one area of the body (e.g., abdomen, thighs, or flanks) and injecting it into the breast to restore volume, shape, and contour.	NCT04273464 NCT05286424 NCT00466765	Autologous Tissue—Uses the patient’s own fat, avoiding synthetic implants and reducing the risk of foreign body reactions or allergies. Natural Look and Feel—Provides a soft, natural texture compared to implants. Minimal Scarring—Only small incisions are needed for fat harvesting and injection. Body Contouring Benefits—Harvesting fat from donor sites (e.g., abdomen, thighs) improves body shape. Low Complication Rate—Fewer major complications (e.g., infection, capsular contracture) compared to implants. Potential for Improved Skin Quality—Fat grafting may enhance skin elasticity and vascularity due to stem cell effects.	Volume Resorption—A significant portion (20–70%) of injected fat may be reabsorbed, requiring multiple sessions. Need for Multiple Procedures—Patients often require 2–3 sessions to achieve desired volume. Fat Necrosis and Oil Cysts—Uneven fat survival can lead to lumps, calcifications, or necrosis. Interference with Mammography—Fat necrosis and microcalcifications may mimic or obscure breast cancer detection. Limited Volume per Session—Only small amounts of fat can be safely grafted at once to ensure vascularization. Donor Site Morbidity—Potential for contour irregularities, pain, or bruising at the liposuction site.

An actively developing method of breast reconstruction based on the use of implants is the technology of using three-dimensional scaffolds; 3D degradable scaffolds are biocompatible, porous structures designed to support tissue regeneration in breast reconstruction by mimicking the extracellular matrix (ECM). They provide a temporary framework for cell attachment, proliferation, and new tissue formation, ultimately restoring breast volume and shape. The key differences between 3D degradable scaffolds and traditional implants are presented in Table 2. An important feature of 3D degradable scaffolds compared to traditional implants is their unique architecture that promotes tissue regeneration in the body, and only maintains shape as in the case of traditional implants.

**Table 2:** Comparison of three-dimensional scaffolds and traditional breast reconstruction methods.

Feature	Three-Dimensional Degradable Scaffolds	Traditional Implants
Material Composition	Biodegradable polymers (PCL, PLGA, collagen), hydrogels, or decellularized ECM [12]	Silicone elastomer, saline-filled shells [14]
Structural Design	Highly porous, interconnected architecture mimicking ECM [27,28]	Solid or fluid-filled, non-porous [16]
Degradation and Lifespan	Gradually degrades as native tissue regenerates [29,30,31]	Permanent or requires replacement after 10–20 years [32]
Host Tissue Integration	Promotes cell infiltration, vascularization, and tissue regeneration [33]	Often leads to fibrous encapsulation (capsular contracture) [34,35]
Customization	Patient-specific shapes via 3D printing/bioprinting [36]	Limited to pre-made sizes/shapes [14]
Mechanical Properties	Designed to match native breast tissue stiffness [36]	Often stiffer, leading to palpability/unnatural feel [14]
Tissue regeneration	Directed formation of tissues with adaptive architecture. This allows for a more natural result, closer to the natural anatomy and function of the mammary glands [37]	Regeneration is mainly aimed at healing wounds and organizing existing tissues [38]
Biocompatibility	Provide more opportunities to control the biointegration process and create tissue that is as close as possible to natural breast tissue. When using 3D degradable scaffolds, biocompatibility is achieved by choosing materials that stimulate tissue regeneration, provide controlled biodegradability, and minimize the immune response [39]	Postoperative complications such as postoperative infection and/or implant rejection may often occur [40]. Scientific articles have described serious cases of “Breast Implant Illness, BII” [41]
Cell differentiation	Three-dimensional degradable scaffolds are a tool for controlling cell differentiation. They create a controlled 3D microenvironment that mimics the natural spatial environment of cells and directs their differentiation into specific cell types [42]	In traditional breast reconstruction methods, unlike the 3D degradable scaffold approach, there is no direct control over cell differentiation. However, the processes associated with tissue regeneration and adaptation certainly affect the cellular composition and organization of the reconstructed mammary gland [43]
Personalized solutions	Three-dimensional printing allows for the creation of individual scaffolds according to the patient’s needs [44]	Actual results may vary depending on individual patient characteristics, surgeon skills, and materials used [45]
Possibility of integrating therapies	Possibility of adding growth factors, medicinal substances to the scaffold [46]	Limited, almost impossible [47]

**Figure 1:**
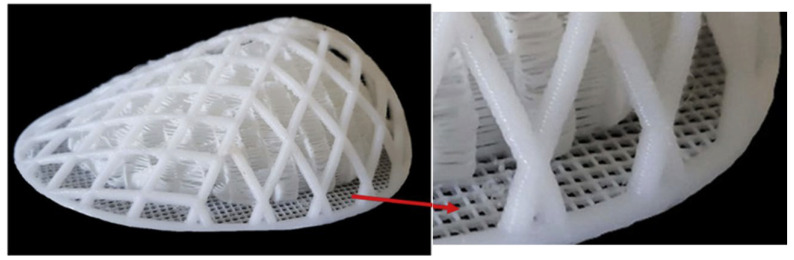
Real images of 3D disassemblable scaffolds for breast reconstruction. A scaffold consisting of two independent structural components. The breast scaffold—comprising an external and internal structure with independently optimized mechanical and structural properties—was fabricated via fused filament fabrication (FFF) using medical-grade polycaprolactone (mPCL). The external structure is designed to withstand biomechanical loads while minimizing stress transfer to developing tissue. The internal structure promotes tissue formation by providing an architecture and mechanical properties tailored to native soft tissue regeneration. Modified from “Additive biomanufacturing of scaffolds for breast reconstruction” by Mohseni M. [52].

Three-dimensional scaffolds can be divided into three broad categories: individual scaffolds based on biodegradable materials; three-dimensional absorbable mesh scaffolds, an example of which is the Lotus design used in combination with autologous fat grafting, which provide consistently satisfactory breast shape and size with no reports of oil cysts, calcifications, or serious complications; and the last category—modular scaffolds incorporating knitted meshes, electrospun nanofibers, or triply periodic minimal surface geometries, which achieve high cell seeding efficiency (up to 92%), promote the formation of vascularized tissue (with a significant increase in the length of vessels and connections in animal models), and support the development of adipose tissue (up to 81.2% of adipose tissue within 24 weeks) [38,48,49,50]. A special feature of modular scaffolds is the possibility of their partial or complete removal after fulfilling the initial function. A striking example here is the patented technology of BellaSeno (WO2016038083A1 [51]) of a three-dimensional scaffold of a biodegradable polymer with magnetic or soluble elements for the formation of voids, imitating the natural structure of tissue (e.g., mammary gland adipose tissue) for optimal vascular ingrowth or filling with cells with the possibility of layer-by-layer removal of spatial structures using specialized instruments (e.g., magnetic probes). The company announced the start of two clinical studies using this technology, but there is no open data on the results or real images illustrating the features of the prosthesis. An example of a disassemblable scaffold is presented in Figure 1. The scaffold has two independent structural components—external and internal. The first provides biomechanical stability, while the structure provides a large pore and fully interconnected pore architecture to facilitate tissue regeneration [52].

## 3. Principles of Tissue Engineering and the Role of Scaffolds

Bioengineering, the combination of cellular technologies and biomaterials, is the most promising direction in this field. The primary goal is to develop scaffolds that promote adipose tissue regeneration. Adipose tissue forms the bulk of the mammary gland, particularly in breast cancer patients whose average age is 60–62 years, so scaffold technology plays a crucial role. This approach incorporates cells or tissue into three-dimensional (3D) matrices of natural or artificial origin to provide spatial orientation for future tissue or organ transplantation.

A mammary gland scaffold must meet several key requirements. First, the matrix must be pliable, mechanically strong, porous, and similar in elasticity to natural breast tissue. Second, its structure should closely mimic the extracellular matrix of the mammary gland. Second, the scaffold material should be biodegradable and biocompatible. Third, the biomaterial must support and stimulate cell adhesion, proliferation, and differentiation, especially for mesenchymal stem cells (MSCs) and adipose-derived stem cells (ADSCs). Potential biomaterials for scaffold fabrication include natural polymers, such as gelatin and collagen, and synthetic polymers, such as polycaprolactone, poly(lactic-glycolic acid), and polyethylene glycol. Synthetic polymers offer more stable mechanical properties than natural polymers. Among the synthetic options, biodegradable composites are preferred because they fully degrade, which minimizes chronic inflammatory reactions in the recipient. Thus, these materials serve only as temporary scaffolds to facilitate tissue regeneration. Additionally, scaffolds can enhance adipose tissue regeneration, offering a promising alternative to traditional reconstruction methods.

### Properties of Breast Tissue

A comprehensive understanding of the anatomical structure of the breast is critical for the successful execution of reconstructive procedures. The mammary gland constitutes a complex branched tubuloalveolar system comprising glandular, adipose, and fibrous tissue components. With advancing age, the proportion of these tissues undergoes changes, resulting in variations in the organ’s physical properties [12].

Typically, each breast contains between 15 and 25 lobules of compound glands, which are embedded within adipose and fibrous connective tissue. Each lobule connects to the nipple–areolar complex via its own lactiferous duct. The interlobular spaces are occupied by connective tissue structures, adipose layers, and fibrous elements. The breast’s size and contour are determined by the overlying skin envelope that defines its volume, as well as the amount of adipose tissue surrounding the glandular compartments. Additional structural support and maintenance of the breast’s anatomical shape are provided by fibrous suspensory ligaments, commonly referred to as Cooper’s ligaments.

Breast reconstruction following mastectomy involves the restoration of the lost volume of skin and soft tissues to recreate an anatomically and aesthetically accurate breast contour. Throughout a woman’s life, the tissue composition of the breast undergoes multiple changes, primarily associated with periods of puberty, menstrual cycle fluctuations, pregnancy, and menopause. These processes are hormonally mediated and influence the breast’s volume, shape, and the elasticity of its supporting ligaments. It is important to note that younger women typically exhibit higher breast tissue density, reflecting the predominance of glandular and ductal components over adipose tissue [52].

A key characteristic of breast tissues is their pronounced nonlinear mechanical response at high levels of deformation. The study by Krouskop T. A. et al. 1998 demonstrated that adipose tissue exhibits an almost constant elastic modulus within the tested deformation range, whereas the elastic modulus of other tissue components varies depending on the magnitude of mechanical loading [40]. According to these findings, fibrous breast tissue displays stiffness values 10 to 100 times greater than those of adipose tissue. Furthermore, normal glandular tissue possesses an elastic modulus comparable to that of adipose tissue at low deformation levels; however, its stiffness increases by approximately an order of magnitude relative to adipose tissue as deformation intensifies.

Pathological alterations markedly affect the mechanical properties of breast tissues, with the stiffness of cancerous tissue under compression exceeding that of healthy tissue by approximately an order of magnitude [41,42]. The linear attenuation coefficient of X-ray radiation by breast tissue is a well-characterized parameter; however, its value varies depending on the tissue composition, which correlates with age-related changes [43]. At energies typical for mammography (approximately 18 keV), the average linear attenuation coefficients for fibrous and adipose tissues are 1.03 cm^−1^ and 0.56 cm^−1^, respectively [44].

Mechanical properties of breast tissues are frequently characterized in the scientific literature using linear elastic Young’s moduli to quantitatively assess tissue stiffness. Experimental approaches commonly involve indentation testing with a punch on small ex vivo tissue samples obtained from larger surgical excisions [45]. This method entails localized compression of a limited tissue area using a cylindrical indenter, typically around 5 mm in diameter, rather than compression of the entire tissue surface. Reported values of Young’s modulus vary considerably across studies: at approximately 1% strain, values range from 3 to 18 kPa, increasing to 57–272 kPa at higher strain levels of 15–20% [40,45,46]. These discrepancies are likely attributable to methodological differences and heterogeneity of the tissue samples used. 

Imaging techniques represent valuable modalities for detecting tissue abnormalities, offering the advantages of non-invasiveness and relative procedural simplicity. These approaches typically involve the application of controlled mechanical stress or excitation to the tissue, followed by assessment of its response to the imposed stimulus. Commonly employed modalities include mammography, magnetic resonance imaging (MRI) and magnetic resonance elastography (MRE), positron emission tomography (PET), computed tomography (CT), breast-specific gamma imaging (BSGI), among others [47].

In a study by Lawrence A. J. et al. 1998 employing magnetic resonance elastography (MRE), the stiffness of glandular tissue was measured at 2.45 ± 0.2 kPa, whereas adipose tissue exhibited a stiffness of 0.43 ± 0.07 kPa [53,54]. Using a similar technique, Hawley et al. (2017) compared the stiffness of dense breasts (characterized by a predominance of fibroglandular tissue) and non-dense breasts in healthy volunteers, reporting mean stiffness values of 0.92 and 0.83 kPa, respectively [55].

Thus, a comprehensive understanding of breast tissue morphology and biomechanical properties is essential for the effective planning and execution of reconstructive procedures.

From a biomechanical perspective, scaffolds designed for breast tissue engineering should possess a low elastic modulus comparable to that of native breast tissue, while simultaneously providing protection to progenitor cells from excessive external mechanical forces. At the cellular level, adipogenesis is enhanced when cells are cultured on substrates with stiffness approximating that of native tissue, whereas increased substrate stiffness exerts an inhibitory effect on this process [52].

Most current research efforts focus on developing scaffolds that replicate the mechanical properties of the native tissue, considering these properties as critical design parameters for soft scaffold fabrication. However, recent findings emphasize the necessity of engineering constructs capable of withstanding both physiological and external mechanical loads, thereby minimizing the transmission of applied stresses not only to the newly forming tissue but also over prolonged periods, facilitating its proper maturation and remodeling. It is important to note that mechanical stress inhibits adipogenesis, and excessive levels of stress and deformation markedly suppress adipose tissue formation [56,57].

In summary, a comprehensive investigation of the mechanical characteristics of breast tissues supports the advancement of prosthetic reconstruction techniques and the development of novel biomaterials that closely emulate the properties of natural tissues, ultimately aiming to achieve optimal functional and aesthetic outcomes for patients.

## 4. Types of Materials for Scaffold Reconstruction

There are two categories of biodegradable polymers. The first category includes biodegradable polymers of natural origin, such as polysaccharides (e.g., alginate, chitosan, and hyaluronic acid derivatives), proteins (e.g., collagen, fibrin, gelatin, and silk), and various types of natural fibers. The second category includes synthetic analogs such as polylactic acid (PLA), polyglycolic acid (PGA), poly(lactic-co-glycolic acid) (PLGA), and polycaprolactone (PCL) [58].

The selection of a biocompatible and bioresorbable material is a critical factor in scaffold design. Rigid polymeric materials provide essential mechanical strength, including resistance to compression and torsion. In contrast, soft composites, particularly hydrogels, are more conducive to cellular proliferation and tissue formation [59].

The effectiveness of tissue-engineered constructs largely depends on the balance between the degradation rate of the polymer and the metabolic activity of the resident cells, as well as on the polymer matrix’s ability to support cell adhesion and spatial distribution [12]. In addition to matching the mechanical stiffness of the target tissue, material selection is also influenced by the polymer’s physical characteristics. It is also influenced by its compatibility with the chosen fabrication technology [60].

### 4.1. Natural Polymers

In addition to synthetic polymer-based scaffolds, biopolymers are used in reconstructive mammary gland surgery [61]. Natural polymers are high-molecular-weight compounds synthesized by living organisms consisting of repeating structural units generated through biological processes [62]. Natural polymers, such as collagen, fibrin, hyaluronic acid, and alginate, have inherent bioactivity. They demonstrate the capacity to support cell adhesion, proliferation, and angiogenesis. This makes them attractive candidates for soft tissue engineering applications.

Hydrogels are promising materials for adipose tissue regeneration. This is due to their high biocompatibility. They also have a minimal pro-inflammatory response. Their mechanical properties can be tuned. These properties closely mimic those of the extracellular matrix [63]. These hydrophilic polymer networks have a high-water retention capacity while remaining insoluble in aqueous environments due to chemical or physical crosslinks. Water penetration into the interchain spaces of the polymer network induces swelling and leads to hydrogel formation [53]. Hydrogels are widely utilized in 3D bioprinting technologies for replicating the architecture and facilitating the regeneration of various human tissues, including skin, vascular structures, neural tissues, cartilage, and adipose tissues, as well as skeletal muscle [64].

Injectable hydrogels can be formed through covalent or chemical crosslinking, utilizing a variety of mechanisms, including Michael addition reactions, click chemistry approaches, enzymatic processes, and photoinitiated polymerization [65,66,67,68]. These hydrogels may be derived from a single polymeric component or designed as hybrid structures that combine biological and/or synthetic polymers.

Hydrogels can encapsulate living cells within three-dimensional scaffolds and be functionalized to deliver growth factors and transmit mechanical cues in a controlled manner. To more accurately mimic the properties of the extracellular matrix, hydrogel structures are modified through the incorporation of bioactive components, including cell-adhesive peptides, protease-sensitive sequences, and molecules that facilitate the binding and release of growth factors [69].

Collagen proteins were one of the first materials [70,71]. Constructions based on them have optimal porosity and adhesive properties. Using collagen hydrogel was especially successful, as it made it possible to obtain a significant amount of newly formed adipose tissue [72,73]. The disadvantage of such scaffolds was low mechanical strength, rapid degradation, and the formation of a pronounced capsule [74,75,76,77]. Kimura et al. 2010 evaluated adipose tissue regeneration in vivo by seeding preadipocytes into collagen sponges with varying biodegradability [78]. The study demonstrated the successful formation of adipose tissue following subcutaneous implantation in mice.

Collagen is a material successfully employed for hydrogel formation. In the study by Huss F. R. M. et al. 2001, a three-dimensional collagen matrix was used to co-culture mammary epithelial cells (MECs) and adipocytes derived from the same donor [70]. The results demonstrated that MECs maintained their characteristic spatial organization and exhibited a growth pattern typical of in vitro conditions under this co-culture configuration.

However, the inherently low mechanical strength of collagen-based scaffolds presents a limitation to their broader utilization [79]. To address this limitation, collagen-based scaffolds can be stabilized through physical or chemical crosslinking techniques, or functionally enhanced by incorporating natural or synthetic polymers, as well as inorganic components.

In a study, Wang X. et al. 2009 developed a co-culture model comprising MCF10A human mammary epithelial cells and pre-differentiated human adipose-derived mesenchymal stem cells (hASCs) [80]. The cells were cultured in a matrix composed of a Matrigel–collagen mixture and seeded onto a three-dimensional porous silk scaffold. The findings revealed that the presence of hASCs led to the suppression of epithelial cell proliferation and promoted the formation of morphologically distinct alveolar- and duct-like structures.

Alginate is a biocompatible material widely used in biomedicine due to its ease of gelation. Alginate-based hydrogels are effective in wound healing, drug delivery, and tissue engineering, as they preserve a structural resemblance to the extracellular matrix of native tissues [81]. Alginate-based scaffolds also had similar properties as collagen scaffolds. Good results were obtained in the regeneration of adipose tissue as well [82]. However, alginate has limitations due to its weak strength characteristics and rapid degradation [76].

Silva P. B. et al. present an advanced three-dimensional in vitro breast tissue model incorporating key components: stromal cells along with their extracellular matrix, and epithelial cells of the parenchyma, assembled into an integrated hybrid system [36]. The model was fabricated using extrusion-based 3D bioprinting of peptide-modified alginate hydrogels, forming a porous structure subsequently seeded with fibroblasts derived from human breast tissue. The developed platform offers a promising in vitro tool for investigating stromal–parenchymal interactions under both physiological and pathological conditions.

Guneta et al. investigated three-dimensional (3D) alginate scaffolds with tunable mechanical and structural properties to evaluate how scaffold characteristics influence stem cell behavior and extracellular matrix formation [83].

In addition to collagen- and alginate-based scaffolds, hyaluronic acid has been studied as a potential material for adipose tissue regeneration. Hyaluronic acid scaffolds have also shown pronounced ability to stimulate adipose tissue regeneration and form a fairly homogeneous structure with a large amount of intercellular matrix [84,85,86]. Human preadipocytes were seeded onto carriers and implanted into athymic mice. The results confirmed the formation of a vascularized scaffold. However, enhancing adipogenic differentiation remains a key challenge for further in vivo studies [84]. The limitation of the use of such scaffolds in clinical practice is their high cost.

Silk-based implants avoid many of the disadvantages of collagen scaffolds and hyaluronic acid constructions [87]. They are characterized by long-term preservation of shape and size, sufficient porosity, low immunogenicity, and long-term degradation. However, the stability of silk degradation products is still unknown. Attempts have also been made to use gelatin to create scaffolds for breast reconstruction. The advantages of these structures are their lack of toxicity and the ease with which growth factors can be added [85,86]. The disadvantages are rapid degradation, low strength, and the necessity of combining them with other materials.

In a separate study by Varma D. M. et al., a plant-derived injectable filler based on carboxymethyl cellulose (CMC) was characterized with the aim of developing a safer and more cost-effective alternative to existing soft tissue augmentation materials [88]. To produce stable hydrogels, a methacrylated derivative of CMC was synthesized and subsequently covalently crosslinked using a redox-initiated system at macromer concentrations ranging from 2 to 4 wt/vol%. The findings indicate that the physicochemical properties of redox-crosslinked CMC hydrogels can be precisely modulated by altering the synthesis conditions, underscoring their promise as a versatile platform for soft tissue engineering applications.

Hydrogels are widely used not only as volumetric fillers for breast reconstruction and scaffold structures in tissue engineering, but also as platforms for drug delivery, including compositions with stem cells and growth factors aimed at stimulating tissue regeneration. In the study by Fu Z. et al., an implantable controlled delivery system was developed based on an enzyme-sensitive hydrogel loaded with sunitinib nanoparticles (NSMRH) [79,89]. The release profile demonstrated the hydrogel’s ability to provide sustained release of the nanoparticles. In vitro functional assays showed that the combined application of NSMRH and ionizing radiation led to significant inhibition of tumor cell proliferation, migration, and invasive potential.

In another study by Jaiswal C. et al., an injectable hydrogel based on silk fibroin was developed without the use of chemical crosslinking agents [90]. This material exhibited minimally invasive properties and was suitable both for the eradication of tumor cells and for the induction of adipose tissue regeneration in breast reconstructive procedures. The application of this system enabled controlled and sustained release of doxorubicin and dexamethasone, maintaining the therapeutic activity of the anticancer agent for up to three weeks. The obtained results confirm the potential of silk-based hydrogels as platforms for the encapsulation of various anticancer compounds, regenerative molecules, and stem cells, while preserving their functional activity for clinical applications.

One of the key parameters in the development of optimal materials for soft tissue substitution is their mechanical performance. Despite the pronounced bioactivity inherent to hydrogels based on natural polymers, such systems generally exhibit limited tunability of mechanical properties compared to their synthetic counterparts [91,92].

Functionalized gelatin methacryloyl (GelMA) represents a hydrogel that has been extensively investigated for human adipose tissue regeneration. GelMA is a semi-synthetic hydrogel obtained by modifying gelatin with methacrylamide and methacrylate groups. In a study by Pepelanova I. et al., the feasibility of synthesizing GelMA hydrogels with a defined degree of functionalization was demonstrated for their use as a three-dimensional platform for cell culture [93]. The primary focus was the development of hydrogel matrices that provide a favorable microenvironment for human adipose-derived mesenchymal stem cells (hAD-MSCs), supporting their adhesion, spreading, and proliferation.

In the study by Zhang J. et al., a composite hydrogel scaffold was fabricated via 3D printing, in which GelMA served as the structural biomaterial, while calcium silicate bioceramic (CaSiO_3_, CS) functioned as the bioactive component promoting adipose tissue regeneration [94]. The in vitro experiments demonstrated that the printed scaffolds not only allow for precise structural customization, but also promote adipogenesis and angiogenesis, highlighting their potential for personalized breast tissue reconstruction.

A low-density porous hydrogel made from GelMA biomaterial was fabricated using indirect additive manufacturing based on layer-by-layer deposition. These 3D-printed structures facilitated the formation of an interconnected porous network that promoted cell attachment to the scaffold surface, with a low level of cell death observed [95].

Another method for obtaining GelMA hydrogel was proposed by Zhu D. et al., where gelatin methacryloyl-based hydrogel (f-GelMA) was produced using a combination of gas foaming and light curing [85]. The resulting biomaterial demonstrated biocompatibility and promoted neovascularization. To enhance adipogenic differentiation, 2D cultures of adipose-derived stem cells (m-ADSCs) were replaced with 3D spheroids (s-ADSCs). The combined use of f-GelMA and s-ADSCs provides a favorable microenvironment for cell survival, migration, and adipogenesis in vitro, while also effectively stimulating the formation of vascularized adipose tissue in vivo.

The properties of a scaffold are determined not only by the type of material used, but also by the processing technology. It is well established that different fabrication methods yield three-dimensional porous structures with varying characteristics, including porosity and interconnectivity, mechanical strength, surface properties, and levels of biocompatibility [86]. Despite their numerous advantages, natural polymers present several limitations that hinder their widespread application in tissue engineering. One of the major drawbacks is their inherently low mechanical strength, which restricts their use in the regeneration of load-bearing tissues [96]. Moreover, many natural polymers—such as fibrin and hyaluronic acid—undergo rapid and often uncontrolled degradation in vivo, which may not align with the temporal requirements of tissue regeneration [97].

### 4.2. Synthetic Polymers

Synthetic polymers are widely used in tissue engineering due to their capacity for precise modulation of physical and chemical properties, including mechanical strength, biodegradation rate, hydrophobicity, and biocompatibility, depending on the intended application [61]. Compared to their natural counterparts, synthetic polymers exhibit more stable mechanical performance over extended periods. Their structural versatility and high degree of parameter control enable the fabrication of materials with tailored porosity, optimized surface characteristics, and predictable degradation kinetics, while also ensuring low batch-to-batch variability [98,99].

Among synthetic biodegradable polyesters, PGA and PLA are the most widely used and thoroughly characterized materials, successfully applied both in general biomedicine and tissue engineering [100]. PGA is distinguished by its high crystallinity, elevated melting point, and low solubility in organic solvents. In contrast, PLA exhibits greater hydrophobicity due to the presence of a methyl group, which reduces water absorption and increases resistance to hydrolysis by creating steric hindrance to the ester bond. As a result, PLA demonstrates slower degradation and better solubility in organic solvents compared to PGA [101]. Copolymers of these monomers (PLGA) can be synthesized with tunable physicochemical properties by adjusting the monomer ratio, making them versatile biodegradable matrices for a variety of tissue engineering applications [102].

Gradwohl M. et al. conducted a comparative evaluation of the technological compatibility of the medical PLGA with an 85:15 monomer ratio using two additive manufacturing techniques: fused filament fabrication (FFF) and direct pellet printing [102]. The aim of the study was to identify the approach that most effectively preserves the physicochemical properties of PLGA. The results indicated that FFF technology, when combined with ethylene oxide sterilization, is the preferable method for producing PLGA-based medical devices.

In the study conducted by Jordao A. et al., the efficacy of a medical scaffold fabricated from another copolymer—poly(L-lactide-co-ε-caprolactone) (PLCL)—was evaluated for its ability to support adipose tissue and stimulate angiogenesis [95]. Using fused deposition modeling 3D printing technology, a biodegradable scaffold with a porous architecture and interconnected pore structure was created, enabling sufficient diffusion of oxygen and nutrients. The obtained results demonstrate the potential of such PLCL-based scaffolds to enhance adipose graft volume retention and improve vascularization, offering a promising strategy for the reconstruction of soft tissue defects.

Additionally, other synthetic polymers, such as polycaprolactone (PCL), are also employed in scaffold fabrication, including in breast tissue reconstruction. PCL, also classified as an aliphatic polyester, is a semicrystalline material with a low melting point and high solubility in organic solvents [103]. Its degradation occurs significantly more slowly than that of PLA and PGA, making it a promising candidate for use as a matrix for sustained drug delivery [104,105].

One of the key challenges in using ADSCs in combination with three-dimensional scaffolds remains achieving clinically meaningful regeneration of adipose tissue in large volumes. In a preclinical animal study, the concept was validated that prevascularization and delayed lipoinjection can support the restoration of substantial volumes of adipose tissue. Using additive biomanufacturing, customized scaffolds were designed and fabricated from medical-grade polycaprolactone (mPCL). The delayed fat injection approach enabled the formation of a vascularized and fibrous tissue bed within the scaffold structure. The subsequent introduction of the adipose graft into the prevascularized scaffold ensured stable integration without signs of necrotic changes. Additionally, it was demonstrated that the use of scaffolds made from slowly biodegradable polymers such as mPCL maintains the required mechanical properties over a time period sufficient to initiate and sustain tissue regeneration processes at a large scale [106].

As part of developing a scaffold for breast reconstruction with customizable properties, Mohseni M. et al. created a biocompatible construct comprising external and internal components with independently optimized mechanical and structural characteristics [52]. The scaffold was fabricated using FFF with medical-grade polycaprolactone (mPCL). The design of the scaffold takes into account both biomechanical and biological requirements, enabling its use in the restoration of medium to large tissue volumes. The construct consists of two independent components: the external shell provides the necessary mechanical stability, reducing stress on the newly forming tissue, while the internal component features a highly porous architecture with fully interconnected pores that promote tissue regeneration. A method for parametric optimization and 3D printing of the outer layer was developed and implemented based on predefined biomechanical properties. The inner component was designed with a gradient pore and channel system to ensure efficient delivery and retention of lipoaspirate.

By combining scaffolds with fat injection, Bao W. et al. fabricated polycaprolactone scaffolds using 3D printing and subsequently loaded with lipoaspirated fat [107]. The constructs were implanted into the subcutaneous tissue of immunodeficient (nude) mice. The results demonstrated that such scaffolds provided the necessary mechanical stability, reducing skin tension and, consequently, interstitial pressure, which in turn improved tissue trophism and supported the formation of vascular structures. Thus, the use of scaffolds helped to reduce hypoxia at the transplantation site and promoted vascularization, thereby enabling prolonged retention of fat graft volume. These findings support the feasibility of using scaffolds with optimized biodegradation rates and additional angiogenic stimulation to enhance the efficacy of fat graft augmentation.

In the study by Zhu X. et al., PCL scaffolds were fabricated using fused deposition modeling [108]. To assess their mechanical properties, a numerical compression model was developed to analyze stress distribution and determine the elastic modulus. The results demonstrated good agreement between the numerical predictions and the outcomes of physical tests. It was found that the elastic modulus of the fabricated structures ranged from 0.08 to 2 MPa, which is considered a favorable range for stimulating breast tissue proliferation.

Spherical three-dimensional matrices made of PCL were developed and printed, followed by implantation into laboratory animals. Three variants of matrices were used in the experiment: (1) made of pure PCL, (2) made of PCL with added collagen, and (3) made of PCL supplemented with a fragment of rat mammary gland tissue. The resulting structures demonstrated high compressive strength and the ability to recover their shape. Histological analysis conducted six months after implantation revealed that matrices containing collagen promoted more active formation of both adipose and fibrous tissue compared to matrices containing autologous tissue. The degree of inflammatory response did not differ between the experimental groups [109].

In a recent study by Cheng M. et al., various therapeutic approaches were compared, including immediate and delayed adipose tissue grafting, as well as the use of platelet-rich plasma [100]. Tissue regeneration of the mammary gland was achieved through the implantation of biodegradable scaffolds, produced by additive manufacturing and filled with autologous adipose tissue grafts. The preclinical evaluation was conducted on a large animal model using 100 mL scaffolds made from medical-grade polycaprolactone (PCL). After 12 months, all experimental groups demonstrated sustained retention of soft tissue volume, averaging 60.9 ± 4.5 mL. No signs of capsular contracture or any early or delayed postoperative complications were observed. 

## 5. Mechanisms of Scaffold Decomposition

The mechanisms of scaffold decomposition include physical degradation and biological recolonization by various cell types, occurring over time frames ranging from months to years. Scaffold decomposition in breast reconstruction involves both physical degradation and chemical–biological processes.

### 5.1. Physicochemical Processes of Biodegradation

**Figure 2:**
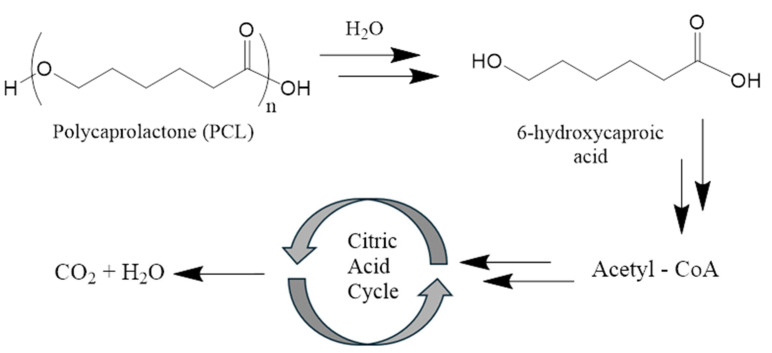
Mechanism of the biodegradation of polycaprolactone.

Commonly used and medically approved 3D degradable scaffold materials, PCL and alginate, have been shown to be biocompatible with surrounding tissues. PCL degrades by hydrolysis to 6-hydroxycaproic acid (fatty acid) and its oligomers. 6-hydroxycaproic acid can enter the citric acid cycle (Krebs cycle) and be excreted from the body after complete decomposition to CO_2_ and H_2_O (Figure 2) [101].

In a 60-month clinical study, PCL scaffolds degraded progressively (54% at 12 months, 92.76% at 60 months) with no reports of necrosis, ischemia, or severe inflammation [38]. Histologic analysis revealed fibrous connective tissue, blood vessels, and fibroblast growth around the scaffold, indicating normal healing. A unique feature of the use of polycaprolactone is the ability to control the degradation rate, which prevents the sudden release of by-products such as caproic acid, minimizing acidity or mechanical stress. This ensures uniform tissue ingrowth and replacement of the scaffold with the natural extracellular matrix [110].

Alginate degrades to non-toxic saccharides. Alginate is inherently non-degradable in mammals due to the absence of the specific enzyme (i.e., alginase) required to cleave its polymer chains. However, ionically crosslinked alginate hydrogels can undergo dissolution through the release of divalent crosslinking ions into the surrounding medium, primarily as a result of ion-exchange reactions with monovalent cations such as sodium ions. Studies combining alginate with polydopamine found no adverse effects in mice, and the scaffolds supported mammary epithelial cell proliferation and reduced tumor size [44].

In vitro, PLA scaffolds lose ~80% of their mass after 8 months due to hydrolysis of ester bonds to monomers and oligomers of lactic acid, leading to acidification of the environment and even greater hydrolysis [111].

**Figure 3:**
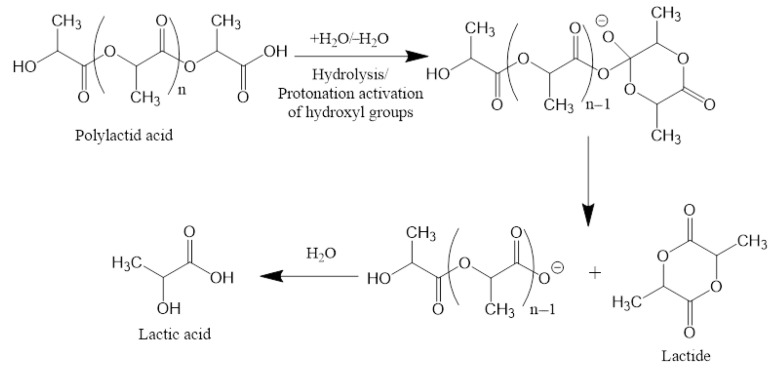
Mechanism of the biodegradation of polylactic acid.

Lactic acid is then metabolized intracellularly to CO_2_ and H_2_O, and excreted through respiration and urine without toxic accumulation (Figure 3) [112]. Thus, the only side effect of PLA scaffold degradation is a decrease in local pH, but the body has mechanisms for rapid response to such a condition.

For scaffolds based on proprietary surgical meshes consisting of a copolymer of glycolide, lactide, and trimethylene carbonate (TIGR Matrix (Novus Scientific Pte. Ltd., Singapore), SERI silk (Allergan, Inc., Medford, MA, USA), and poly-4-hydroxybutyrate (PHASIX (CR Bard, Inc./Davol Inc., Warwick, RI, USA)), mesh resorption with gradual replacement by body tissues was demonstrated [113]. During the biodegradation of poly-4-hydroxybutyrate (P4HB), the primary degradation product is 4-hydroxybutyrate (4HB), an endogenous metabolite naturally generated in the human body through the metabolic pathway of γ-aminobutyric acid (GABA). The existence of this physiological catabolic route facilitates efficient processing and elimination of degradation by-products, thereby ensuring the high biocompatibility of P4HB and contributing to a minimal inflammatory response [30,114]. Implantable mesh constructs such as PHASIX^®^ and other P4HB-based devices designed for soft tissue repair undergo complete biological degradation and resorption within 12 to 18 months following implantation [115].

Current data suggest that degradation products from 3D-printed breast scaffolds—primarily PCL and alginate derivatives—are well tolerated and support tissue regeneration without significant toxicity. Minor volume loss due to scaffold degradation is the main adverse effect observed, which is controlled by gradual degradation rates. However, long-term follow-up is required to assess long-term effects.

### 5.2. Effect of Decomposed Products on Surrounding Tissues

Among the effects of scaffold degradation products on surrounding tissues, one can highlight the influence on cellular viability in nearby tissues and activation of local immunity. PCL is hydrolyzed to caproic acid—a saturated medium-chain fatty acid with six carbon atoms, metabolized in the liver via β-oxidation in mitochondria [116]. In vitro studies on four cancer cell lines (HCT-116 (human colorectal carcinoma cells), A-431 (human skin epidermoid carcinoma cells), CCD-33Co (normal human colon fibroblasts), and MDA-MB-231 (human mammary gland adenocarcinoma cells)) showed that caproic acid in goat milk led to a decrease in the viability of cancer cells compared to the control group, including through increased regulation of genes (P21 (cyclin-dependent kinase inhibitor 1)) involved in apoptosis [117]. However, a clinical study by Roopashree and colleagues found no significant difference in caproic acid levels between breast cancer patients and healthy controls [116].

It is worth mentioning separately scaffolds loaded with drugs [118]. In vitro studies using MCF-10A breast cancer cells and an animal model using immunocompromised female nude *Foxn1nu* mice subcutaneously injected with MDA-MB-231 cells demonstrated the potential of scaffolds composed of PLGA, polyethylene glycol (PEG), and PCL loaded with prodigiosin (PG) or paclitaxel for local drug release and thus suppression/treatment of locally recurrent triple-negative breast tumors. A similar effect was achieved when 3D degradable scaffolds based on gelatin with methacryloyl groups (GelMA), and pectin loaded with paclitaxel and cyclodextrin complexes were investigated on the pathogenesis of triple-negative breast cancer in vitro and in vivo [119].

An interesting approach using multicomposite 3D polycaprolactone (PCL) scaffolds with multifunctional bioactive ceramics, cobalt orthosilicate (Co_2_SiO_4_, CoSi), was proposed by Jupei Zhang. In in vitro studies on 3T3-L1 preadipocytes, the researchers noted high proliferative activity of cells on cobalt-containing scaffolds. In the presence of CoSi scaffolds, they drew attention to the high migration and tube-forming ability of human umbilical vein endothelial cells (HUVECs) [120]. The effect of the scaffolds on tumor development was evaluated in vivo using a subcutaneous breast cancer model in nude mice. After irradiation of mice with a cobalt scaffold, the tumor size gradually decreased until it was completely undetectable after 14 days.

Thus, in vitro and in vivo studies have shown the biocompatibility and safety of using 3D degradable scaffolds based on polycaprolactone, alginate, and polylactic acid; however, the long-term effects of their use remain poorly studied.

### 5.3. Time During Which Scaffolds Decompose and How This Affects the Healing Processes

Studies indicate that healing processes follow a time-dependent course; 3D degradable scaffolds degrade over varying time frames from hours to years, with optimal healing occurring when degradation aligns with the three main healing stages: initial cell attachment (0–2 weeks), matrix formation (2–8 weeks), and tissue maturation (beyond 8 weeks). Early degradation (0–2 weeks) permits initial cell attachment and mild immune response; intermediate stages (2–8 weeks) support extracellular matrix deposition and angiogenesis; and later stages (beyond 8 weeks) correlate with mature tissue formation [31]. The degradation time of 3D-printed breast scaffolds and its impact on the healing process depends on the scaffold material and degradation kinetics. For PCL, complete degradation has been shown to typically occur within 2–4 years in vivo, depending on molecular weight and scaffold design [121]. However, degradation of PCL over several years provides stable structural support, allowing tissue to ingrow uniformly and replace the extracellular matrix [33].

The process of tissue repair using scaffolds is related not only to the material and decomposition products, but also to the pore size [27]. Scaffolds with 300–500 μm pores mimic native adipose tissue architecture, promoting fat graft survival and reducing necrosis by 40% in preclinical models [28].

Therefore, when choosing a material for breast reconstruction, it is necessary to pay attention not only to the chemical formula of the polymer used to form the main scaffold, but also to the internal structure of the 3D degradable scaffold, the size of the pores, additional coatings, and the possibility of using the scaffold, including for therapeutic purposes. However, it should be remembered that any polymer is subject to degradation processes and the reaction of each organism is individual, even to biocompatible materials.

## 6. Preclinical and Clinical Studies of 3D Degradable Scaffolds

### 6.1. Preclinical Studies of the Use of 3D Degradable Scaffolds

Initially, the safety and effective transplantation of scaffolds, as well as adipose tissue regeneration, angiogenesis, tissue ingrowth, and preservation of mechanical and structural properties are investigated in animals and in vitro. Both rodents (nude mice and rats) and large animals such as pigs are used in the studies [34]. In the in vitro work, based on mechanical indicators such as the ability to maintain shape under strong dynamic pressure from body movement, an optimal design was selected that replicates the natural shape of the breast [122]. It was a polycaprolactone mold with a 200 μm radial thread and a 45° spoke structure. The ability of this design to be colonized by fat cells, to maintain its shape inside the body, and to be safe was tested in a rat model. Autologous inguinal fat from Sprague-Dawley rats was used to populate the 3D shape. When evaluating the thickness of the fibrous capsule, no significant differences were found compared to a standard silicone implant. Based on the results obtained, the authors suggested that the use of the 3D structure they developed, including a carrier in the form of a silicone implant, will allow the shape and volume of adipose tissue to be preserved for as long as possible. A special feature of this work is the use of autologous animal fat without an additional cultivation step.

To better understand how the implant will behave in the human body, medium and large animal models, such as pigs, are used for research [123]. Thus, 3D degradable scaffolds in the form of zigzag and fullerene-type polycaprolactone beads with 0.2 mm thick sections with or without collagen treatment for faster adipose tissue regeneration were investigated. At 3 months, the collagen-coated polycaprolactone spherical scaffold (PCL-COL Sphere) had more collagen fibers and lower levels of the pro-inflammatory cytokines TNF-a and IL-6.

A peculiarity of humans compared to animals is the need to replace a significant volume of adipose tissue; therefore, when it is necessary to obtain it on a large scale, adipose tissue engineering technology is used. The group of Chhaya [124] proposed an approach to breast reconstruction with a 3D degradable scaffold based on poly(d,l)-lactide polymer with a pore size > 1 mm, initially seeded with human umbilical cord perivascular cells and, after 6 weeks of cultivation, seeded with human umbilical vein endothelial cells. Angiogenesis and adipose tissue formation were evaluated in the athymic rat model. After 24 weeks, the amount of adipose tissue increased to 81.2% and the endothelial cells formed a functional capillary network. Delayed cell injection adipose tissue engineering technology coupled with a patient-specific polycaprolactone scaffold in an immunocompetent pig model demonstrated angiogenesis and adipose tissue regeneration [34]. The possibility of using autologous adipose tissue scaffolds to augment the soft tissue shell of reconstructed breasts was demonstrated by the group of Maurizio Verga in a series of 24 patients [125]. After fat collection using the Coleman technique (collection was performed using a hand syringe with manual suction and a 3 mm blunt cannula, followed by filtration through a metal sieve and rinsing with saline), it was shaped using fibrin glue and applied to the pectoral muscle. This approach not only demonstrated a high aesthetic result but also led to a reduced risk of implant-related complications, such as wrinkle formation and visibility.

To address another challenge associated with breast reconstruction, the ability of the scaffold to retain its shape for a long time, a group of researchers proposed using a 3D degradable scaffold based on Poly(ε-caprolactone-co-p-dioxanone) with knitted meshes and electrospun nanofibers, shaped like a bra. In vitro experiments showed that adding a knitted mesh and a layer of electrospun nanofibers to the scaffold significantly increased the efficiency of cell seeding, cell attachment, and proliferation compared to the 3D-printed scaffold alone [36].

### 6.2. Clinical Trials of the Use of 3D Degradable Scaffolds

According to the request for “Breast Reconstruction” on the website clinicaltrials.gov, there are 607 registered studies [126]. Only two of them (NCT03348293 and NCT06993714) focus on using three-dimensional scaffold technology for breast reconstruction (Table 3). And only one of these studies has published results. A prospective clinical trial (NCT03348293) included 26 subjects with tumor sizes varying from 1 to 8 cm, who were treated with breast reconstruction surgery using polycaprolactone as the reconstructive material [127]. The design and printing of implants for this clinical study was innovative and based on data obtained from three-dimensional images derived through magnetic resonance imaging performed by the Siemens Trio Tim 3.0T device, followed by processing in Mimics 17.0 software (Materialise, Leuven, Belgium), which enabled 3D reconstruction of the target area. However, an important limitation of this clinical investigation is the absence of a control group that utilized classical methods of breast reconstruction. Among complications observed during follow-up periods, only implantation site depression formation was noted: within one year post-surgery, four women (15.4%) and increasing to seven individuals (26.9%) after two years. Patient satisfaction and quality-of-life scores following the procedure were recorded as follows: 68.5 ± 15.7 points at six months, 65.4 ± 14.2 points at twelve months, and 62.8 ± 15.9 points at twenty-four months. The average degradation rate of the used scaffolds amounted to 54.07% at 12 months, 74.48% at 24 months, 86.94% at 36 months, 87.36% at 48 months, and 92.76% at 60 months.

A follow-up clinical trial (NCT06993714) has been initiated and is currently recruiting participants to address the shortcomings identified in the initial study (NCT03348293). It is planned to enroll 120 individuals into three treatment groups: the experimental group will undergo breast reconstruction using 3D-printed biodegradable breast implants, while the two control groups will receive either conventional organ-sparing surgery or breast reconstruction with silicone implants. Implant modeling will be based on images acquired via simple scanning and 3D dynamic contrast scanning magnetic resonance tomography, processed using MIMICS 17.0, Geomagic, and 3-matic software (Materialise, Belgium). The authors specify that selective laser sintering technology will be employed for printing the scaffold. Polycaprolactone is indicated as the material used in this work.

### 6.3. Evaluation of the Effectiveness and Reliability of 3D Degradable Scaffolds

The efficacy and reliability of 3D degradable scaffolds for breast reconstruction are assessed in two ways: patient feedback and determination of the degree of degradation. The most commonly used method for assessing patient-reported outcomes is the quality-of-life questionnaire, BREAST-Q, which measures patients’ satisfaction and quality of life before and after breast surgery. The information obtained in this way about breast satisfaction, psychosocial, physical, and sexual well-being from patients allows evaluation of effectiveness (aesthetic and functional results) and reliability (complication rate, long-term stability) [128]. A retrospective study by Robert D. Rehnke and colleagues demonstrated the safety and efficacy of using a three-dimensional absorbable mesh scaffold (Lotus) combined with autologous fat grafting for breast reconstruction. The study included 22 female patients (average age: 60.5 years) who underwent the procedure. Over a mean follow-up period of 19 months, patients reported satisfaction with the final shape and size of their reconstructed breasts. Mammographic and magnetic resonance imaging (MRI) revealed no evidence of oil cysts or calcifications. Histological analysis further confirmed the absence of capsule formation [49]. In a prospective clinical trial (NCT03348293) evaluating polycaprolactone-based 3D implants, indentation at the implant site was the sole complication observed during follow-up. This occurred in four women (15.4%) within one-year post-surgery and in seven patients (26.9%) by the two-year follow-up [127].

In addition to evaluating the cosmetic effects and engraftment of fat cell-laden scaffolds, it is equally important to assess the effect of autologous fat grafting on the development of future metastases or disease recurrence when evaluating the efficacy of this approach to breast reconstruction after tumor tissue removal. The lack of effect of autologous fat tissue on future metastasis or recurrence has been demonstrated in an animal model and a retrospective human study [129,130]. Immunodeficient Non-obese diabetic (NOD) mice with mutations *Prkdc^scid^* and *Il2rg^tm1Wjl^* (NOD-SCID gamma mice) underwent fat transfer for breast reconstruction with an insignificant number of tumor cells present. No increase in tumor size, proliferation, histologic grade of malignancy, or metastatic spread was observed. A retrospective, population-based cohort study included 4709 patients who underwent breast reconstruction after surgical resection between 2001 and 2018. Patients who received fat grafting were compared with those who did not for metastasis and death within 15 years of reconstruction. Among breast cancer patients who subsequently underwent fat grafting, there was no significant increase in distant metastasis or all-cause mortality compared to patients who did not undergo fat grafting.

## 7. Three-Dimensional Disassemblable Scaffolds in Breast Reconstruction: Current Status and Future Applications

Breast cancer is one of the most common malignant tumors worldwide. The main method of treating this pathological condition is partial or radical mastectomy. Total (radical) mastectomy is still the gold standard of treatment. After any type of mastectomy, approximately 40% of patients resort to the help of a plastic surgeon to correct the resulting defect [131]. Several approaches are used in reconstructive surgery:

- implant-based reconstruction with silicone or saline—filled prostheses;
- autologous (flap) reconstruction which utilizes the patient’s own tissue harvested from donor sites (e.g., abdomen, back, thigh, or buttocks) to create a natural breast mound. Common flap techniques include DIEP flap (from lower abdomen), latissimus dorsi flap (from back), profunda artery perforator flap (posterior thigh), superior/inferior gluteal artery perforator flap (buttocks), lumbar artery perforator flap (love handle area) [69,132].

Usually, a combination of implants and autologous tissues (based on adipose tissue, skin–muscle or skin–fascial flap) is used.

For several decades, acellular dermal matrices (ADMs) have been used for breast reconstruction. They are skin devoid of cells, elements with antigenic activity, and a double-stranded DNA content of less than 50 ng/mg [132]. ADMs can be obtained from both animals and humans, and they contain collagens, elastic fibers, fibronectin, laminin, and hyaluronic acid [133,134,135]. It has been established that such skin matrices are subject to neovascularization and colonization by recipient cells [136,137,138]. In the case of reconstructive surgeries after mastectomy, the use of ADMs improves the skin coverage of the silicone implant or skin expander used [139]. In addition, the use of ADMs allows avoiding such complications as contracture of the gland capsule, as well as necrosis of the alveolar region [35,140,141,142]. When comparing ADMs with 3D disassemblable scaffolds for breast reconstruction, several factors must be considered, including cost, scalability, and complication profiles. ADMs, derived from processed human or animal tissues, have become widely used due to their availability and ability to support soft tissue integration; however, they are often associated with high costs and difficult logistics that can limit scalability. In contrast, 3D disassemblable scaffolds offer the advantages of patient-specific design and potentially lower manufacturing costs as production processes become more mature and standardized. In terms of complications, ADMs are associated with an increased risk of seroma, infection, and delayed wound healing, in part due to their biological origin and immunogenic potential [143]. It has also been shown that ADMs should be used with particular caution in patients with diabetes, obesity, and smokers [144]. Meanwhile, 3D disassemblable scaffolds, especially those developed using biodegradable and bioinert materials, may reduce such risks by minimizing immune responses and allowing controlled degradation in concert with tissue regeneration.

The use of autologous adipose tissue in reconstructive surgery of the mammary gland has a number of positive aspects: low immunogenicity, ease of procedure, low number of complications, good effect [28,145]. In this regard, the use of autologous fat tissue has become standard in the treatment of those patients who do not want to undergo long-term treatment due to complex reconstructive surgeries and also reject the idea of having foreign material in their body. Despite the good cosmetic effect, most studies have found a so-called decrease in the volume of the fat graft, which is observed over time in 30–40% of cases [146].

Several approaches have been proposed to solve this problem. One of these approaches is the addition of PRP to lipoaspirate. Blood platelets are a natural reservoir of growth factors that stimulate tissue regeneration. The use of PRP together with autologous fat tissue leads to the preservation of the graft volume, which has been shown both experimentally and in clinical studies [28,147,148].

Another approach that allows you to preserve the required volume of fat graft is the use of the stromal vascular fraction (SVF) and MSCs of adipose tissue. It has now been shown that the ability to stimulate regeneration using fat tissue transplantation is achieved through adipose tissue MSCs in the SVF [28]. It is the SVF that ensures the survival of adipose tissue due to its angiogenic properties and the production of various growth factors [149]. In this regard, the modern approach involves enriching the fat autograft with the SVF; in addition, it is possible to use MSCs from other sources [28,149,150,151]. At the same time, a clinical study conducted over 1 year established that the use of a SVF-enriched fat autograft leads to better results, including in maintaining the contour of the gland [149,152]. However, patients and doctors remain wary that the introduction of the SVF can stimulate tumor recurrence. At the same time, in all studies, transplantation of SVF-enriched adipose tissue did not lead to an increase in the recurrence rate of breast cancer compared to transplantation of exclusively autologous adipose tissue. These data were obtained in studies conducted over 3 and 5 years [153,154].

Another approach to increasing the survival rate of adipose tissue grafts is the use of so-called cell protectors. One study showed the positive effect of poloxamers [155]. Poloxamers are copolymers of polyoxyethylene (PEO) and polyoxypropylene (PPO) segments in different proportions. It was found that poloxamer 188 (P188) is able to stimulate the restoration of cell membranes, which leads to a decrease in the level of cell death [155,156].

Despite the proposed approaches, reducing the volume of the transplant based on autologous fat tissue remains an urgent problem for reconstructive surgery of the mammary gland. In this regard, one of the solutions may be 3D printing of a mammary gland scaffold [49]. The use of 3D printing for mammary gland reconstruction was first proposed in 2011 by Melchels et al. [157]. To create scaffolds, it has been proposed to use polycaprolactone (PCL), PLA, PLGA, and Poly(alkyl ethylene phosphate)-based (co)polymers, polydioxanone PDO [158,159,160,161,162,163]. The first experimental scaffolds showed promising results [69,164]. However, the resulting tissues were more rigid, as they contained more fibrous connective tissue than fat. The creation of a more elastic scaffold and the use of adipose tissue MSCs made it possible to obtain better results [38,165,166].

One method for making prostheses for breast reconstruction is 3D printing [167]. Currently, several approaches are being used in 3D printing: inkjet, extrusion, laser-assisted, and stereolithography printing [32]. A critical step in this technology is the creation of a three-dimensional model of the breast. Other methods for generating 3D degradable scaffolds include electrospinning, phase separation, gas foaming, particulate leaching, freeze-drying, and decellularization [166,167]. The choice of technique largely depends on the researcher, the material they are working with, and their specific objectives.

Electrospinning is a process where an electrically charged polymer stream in a viscous state or solution is stretched into fibers under the influence of electrostatic forces [168]. Electrospinning is typically used to create scaffolds made of collagen and gelatin nanofibers characterized by high porosity and large surface area [169]. Additionally, polymers such as PLLA (poly(l-lactic acid)), PLGA (poly(lactic-co-glycolic acid)), PGA (poly(glycolic acid)), and PCL (polycaprolactone) may also be utilized. Fiber diameters range from 400 to 1100 nm, porosity varies between 80 and 95%, and cell survival rates reach up to 80%.

The freeze-drying or lyophilization method involves the dehydration of a polymer solution. This approach yields scaffolds with pore sizes ranging from 20 to 200 μm (or alternatively reported as 50–450 nm) and porosities between 30 and 80%. Cell viability reaches up to 90% [170]. The pore size is influenced by freezing frequency, polymer concentration, and temperature conditions [171]. Phase separation is achieved thermally or by adding a non-solvent. It is used for producing porous membranes and foam-like scaffolds. Polymers like PLLA (poly(l-lactic acid)) and PLGA (poly(lactic-co-glycolic acid)) are typically employed. The fiber diameter ranges from 50 to 500 nm, porosity varies between 60 and 98%, and cell viability reaches up to 98%.

Decellularization involves removing all cells from tissue while preserving the natural composition of extracellular matrix components and primarily its architecture. Importantly, the retained matrix must not be toxic to cells but rather facilitate their proliferation and differentiation. Cell removal can be accomplished using surfactants and enzymes. At the final stage, nucleases are generally employed. The absence of cells is confirmed by nuclear staining and DNA concentration measurements.

Most scaffolds designed for breast prosthesis take the form of a hemisphere without considering possible mechanical characteristics of the prosthesis. Several researchers attempted to avoid such drawbacks. Sharon Kracoff-Sella et al. utilized finite element analysis to print a highly porous dome-shaped prosthesis composed of PCL (polycaprolactone) on a 3D printer [172]. The wall consisted of two layers connected by pillars, and the base was absent, allowing direct contact of the material with the recipient’s tissues. Mechanically, the prosthesis closely resembled human adipose tissue.

However, the problem of vascularization of transplanted structures and adipose tissue still remains. In this regard, some authors have proposed a two-stage reconstruction: first, the scaffold is transplanted, and after some time, the lipoaspirate [34]. This approach allowed us to obtain better results in preserving the transplant volume. The use of non-resorbable structures requires repeated surgical intervention. In this regard, another problem with the use of polymer-based scaffolds is the creation of structures with a given resorption time. Currently, implants have been created that completely degrade in 12 and 24–36 months [173,174].

Thus, it is now obvious that the use of autologous adipose tissue is a promising approach in reconstructive surgery of the mammary gland. However, researchers are faced with a number of problems that are united around one task—stimulating the regeneration of transplanted adipose tissue, since this is what prevents the observed decrease in the volume of the operated mammary gland over time. The creation of an optimal scaffold for adipose tissue that would ensure optimal neovascularization and formation of new adipocytes from their precursors is also considered within the framework of this problem. In addition, the possibility of recurrence of breast cancer is still an acute problem for reconstructive surgery. Despite the available data on the absence of a stimulating effect of transplanted scaffolds and adipose tissue on the development of malignant tumors, oncological alertness should always be in the first place when choosing one or another method of treatment [175].

## 8. Conclusions

In recent years, significant progress has been made in breast reconstructive surgery, especially with the use of 3D disassemblable scaffolds. Three-dimensional disassemblable scaffolds are a promising solution that overcomes the limitations of traditional methods. These scaffolds can be adapted to the unique anatomy of the patient and are useful for creating temporary or permanent tissue support. The most promising method for post-mastectomy breast reconstruction involves the use of autologous tissues and cellular components integrated into synthetic or biopolymer scaffolds. Key areas showing great promise include the following:

- Development of new biocompatible and biodegradable polymers, including hybrid materials combining biopolymers with synthetic polymers;
- Advanced 3D printing technologies to create personalized scaffolds that precisely match the patient’s anatomy;
- Integration of scaffolds with cell therapies, particularly stem cells, to improve tissue regeneration;
- Immobilization of growth factors on the surface of scaffolds to stimulate cell proliferation and differentiation;
- Creation of scaffolds with embedded micronetworks to monitor tissue health in real time and guide repair processes.

Despite significant advancements, key challenges must be addressed to translate disassemblable scaffolds into clinical practice. Below, we outline the most pressing gaps and research priorities:

- Imbalance in the rate of tissue regeneration and scaffold resorption.
- Specific priorities include optimizing pore size and architecture to enhance adipogenesis and vascularization.
- Standardized fabrication and implantation protocols are needed to ensure reproducibility and clinical safety. Long-term clinical trials are essential to evaluate functional outcomes, patient satisfaction, and potential complications.
- Development of methods for stimulating sensory feedback and neuronal growth in the area of scaffold implantation.
- Additionally, the integration of new materials, biofabrication methods, and therapeutic strategies will be critical to developing safer and more effective breast reconstruction solutions.

Integrating these approaches and encouraging active collaboration between researchers, clinicians, and industry stakeholders will accelerate the translation of 3D disassemblable scaffolds from experimental models to everyday clinical practice, ultimately improving reconstructive outcomes and quality of life for women after mastectomy.

## Figures and Tables

**Table 3:** Clinical trials of the use of 3D degradable scaffolds.

Clinical Trial Identificator	The Study’s Title	Features of Implants	Number of Participants	Complications	Breast-q Score	Scaffold Degradation Timelines	Published Results Are Accessible
NCT03348293	Safety Study of 3D Printing Personalized Biodegradable Implant for Breast Reconstruction	3D image reconstruction and printing. Magnetic model images data were produced by Siemens Trio Tim 3.0 T MRI. The MRI data were then imported into Mimics 17.0^®^ [Materialise, Leuven, Belgium] for 3D reconstruction of the targeted area. Biologically active material PCL was selected for implant at printing.	26 Only experimental group.	One year after operation, mild depression at the implantation site was observed in four patients (15.4%). This number increased to seven (26.9%) after two years of surgery. No flap necrosis or ischemia was observed in the nipple and areola area in all patients.	68.5 ± 15.7 at 6 months, 65.4 ± 14.2 at 12 months, and 62.8 ± 15.9 at 24 months.	The average degradation rate of 3D-printed scaffolds is 54.07% at 12 months, 74.48% at 24 months, 86.94% at 36 months,87.36% at 48 months, and 92.76% at 60 months.	Yes [48]
NCT06993714	3D-printed Biodegradable Breast Implants for Breast Restoration	The image of the mammary gland model is obtained by simple scanning and 3D dynamic contrast scanning. The software MIMICS 17.0, Geomagic, and 3-matic were used to design an individual 3D model of the mammary gland. The model of the breast implant was printed layer by layer from polycaprolactone using selective laser sintering technology with subsequent sterilization.	120 Three groups: 1. Experimental: Breast restoration surgery based on 3D-printed degradable biological implants; 2. Active Comparator: Traditional breast-conserving surgery; 3. Active Comparator: Traditional silicone prosthesis breast reconstruction.	No information available.	No information available.	No information available.	No information available.

## Data Availability

Data are available in a publicly accessible repository.

## Abbreviations

The following abbreviations are used in this manuscript:

BC	Breast cancer
MSCs	Mesenchymal Stem Cells
ADSCs	Adipose-Derived Stem Cells
PCL	Polycaprolactone
PLGA	Poly(lactic-glycolic acid)
PLA	Polylactic Acid
PGA	Poly(glycolic acid)
FFF	Fused Filament Fabrication
PLCL	Poly(L-lactide-co-ε-caprolactone)
ADMs	Acellular Dermal Matrices
